# Clinical Value of Bone Radiotherapy in a Prospective Cohort of Metastatic Breast Cancer Treated with Anti-CDK4/6 †

**DOI:** 10.3390/jcm14134662

**Published:** 2025-07-01

**Authors:** Edy Ippolito, Lucrezia Toppi, Sofia Carrafiello, Carlo Greco, Michele Fiore, Rita Alaimo, Salvatore Minuti, Francesco Pantano, Giuseppe Casale, Rolando Maria D’Angelillo, Adriana Turriziani, Maria Grazia De Marinis, Sara Ramella

**Affiliations:** 1 Research Unit of Radiation Oncology, Department of Medicine and Surgery, Università Campus Bio-Medico di Roma, Via Alvaro del Portillo, 21, 00128 Rome, Italy; e.ippolito@policlinicocampus.it (E.I.); sofia.carrafiello@unicampus.it (S.C.); m.fiore@policlinicocampus.it (M.F.); salvatore.minuti@unicampus.it (S.M.); s.ramella@unicampus.it (S.R.); 2 Operative Research Unit of Radiation Oncology, Fondazione Policlinico Universitario Campus Bio-Medico, Via Alvaro del Portillo, 200, 00128 Rome, Italy; l.toppi@policlinicocampus.it (L.T.); r.alaimo@policlinicocampus.it (R.A.); a.turriziani@policlinicocampus.it (A.T.); 3 Research Unit of Medical Oncology, Department of Medicine and Surgery, Università Campus Bio-Medico di Roma, Via Alvaro del Portillo 21, 00128 Rome, Italy; francesco.pantano@policlinicocampus.it; 4 Operative Research Unit of Medical Oncology, Fondazione Policlinico Universitario Campus Bio-Medico, Via Alvaro del Portillo, 200, 00128 Rome, Italy; 5 Research Unit Nursing in Palliative Care, Fondazione Policlinico Universitario Campus Bio-Medico, 00128 Rome, Italy; g.casale@policlinicocampus.it (G.C.); m.demarinis@policlinicocampus.it (M.G.D.M.); 6 Radiation Oncology, Department of Biomedicine and Prevention, University of Rome “Tor Vergata”, 00133 Rome, Italy; rolandomaria.dangelillo@ptvonline.it; 7 Research Unit of Nursing Science, Department of Medicine and Surgery, Università Campus Bio-Medico di Roma, Via Alvaro del Portillo, 21, 00128 Roma, Italy

**Keywords:** CDK4/6 inhibitors, bone metastases, radiotherapy

## Abstract

**Background**: CDK4/6 inhibitor plus ET is a standard treatment for advanced HR+ BC. This study evaluates the efficacy and safety of CDK4/6 inhibitors with concurrent RT (SBRT and non-SBRT) in terms of pain, analgesic therapy changes, toxicities, and net clinical benefit (NCB). **Methods**: BC patients with bone metastases treated with RT and CDK4/6 inhibitor in the prospective observational COMBART study were analyzed. Pain was measured with the NRS. The NCB was defined by pain reduction (NRS), toxicity, and treatment changes. Adverse events (AEs) were graded per CTCAE v5.0. Statistical tests included chi-square and *t*-test. **Results**: Forty patients were treated with CDK4/6 inhibitor (palbociclib 30.8%, ribociclib 51.3%, abemaciclib 17.9%) and RT (131 lesions; 100 SBRT, 31 non-SBRT). The mean NRS score dropped from 3.52 (pre-treatment) to 1.31 (post-treatment) (*p* < 0.001), with better outcomes for patients treated with moderate hypofractionation (58.6% vs. 39.9% pain relief, *p* = 0.016). Pain relief was independent of the type of CDK4/6 inhibitor used (*p* = NS). Analgesic reduction was most common with palbociclib (35.4%, *p* = 0.001). Eight toxicities (grade 1–2) were reported. The NCB was 0.6 overall, higher with non-SBRT (0.74 vs. 0.52). **Conclusions**: RT plus CDK4/6 inhibitor, especially with moderate hypofractionation, significantly reduced pain with manageable toxicity. Analgesic therapy can often continue without stopping CDK4/6 inhibitor.

## 1. Introduction

Breast cancer represents the most frequently diagnosed malignancy among women on a global scale. According to data from 2014 to 2020, the overall 5-year relative survival rate across all stages of breast cancer was 91.2%, whereas it dropped significantly to 31.9% in cases presenting with distant metastatic disease [1]. Within the metastatic setting, the outcomes are influenced by multiple factors including patient age, existing comorbidities, functional status, tumor biology, as well as the burden, sites of metastasis, and treatment strategies employed [2]. Bone is the most common site of distant metastasis in patients with metastatic breast cancer. It is estimated that approximately 65% to 75% of women with advanced breast cancer will develop bone metastases during the course of their disease. Particularly, among the various breast cancer subtypes, HR+/HER2− tumors exhibit a higher propensity for bone involvement [3,4].

In patients with HR+/HER2− advanced breast tumors, cyclin-dependent kinase 4/6 inhibitors (CDK4/6 inhibitor), including palbociclib, ribociclib, and abemaciclib, have significantly improved the outcomes with a median PFS ranging from 14 to over 25 months [5,6,7,8,9]. CDK4/6 inhibitors act by targeting cyclin-dependent kinases 4 and 6, which are key regulators of the transition from the G1 to the S phase of the cell cycle. By inhibiting CDK4/6 activity, these agents induce cell cycle arrest, and reduce tumor cell proliferation [10].

Radiotherapy (RT) plays a key role in the management of bone metastases by providing effective pain relief, preventing or treating skeletal-related events (such as fractures or spinal cord compression), and improving overall quality of life. It is commonly used as a palliative treatment in both localized and multifocal bone lesions. Clinical studies have demonstrated that both conventional RT and stereotactic body radiotherapy (SBRT) induce significant pain reduction and enhance skeletal stability [11,12,13].

In the multidisciplinary management of patients with HR+/HER2− advanced breast tumors with bone metastases, the integration of radiotherapy (RT) with CDK4/6 inhibitors remains a clinical challenge, with several aspects yet to be fully clarified. The radiosensitizing effect of CDK4/6 inhibitor, observed in preclinical studies [14,15], is attributed to their ability to inhibit DNA damage repair induced by RT and therefore increasing tumor cytotoxicity. However, this potential synergy may also elevate the risk of hematologic and gastrointestinal toxicity, particularly in combined therapies. Currently, available clinical evidence seems to support the combination of these treatments with a modest increase in toxicity [16,17].

Pain management remains a cornerstone in the treatment of bone metastases. Nonsteroidal anti-inflammatory drugs (NSAIDs) are commonly employed as first-line therapy for mild-to-moderate pain, while minor opioids (e.g., codeine and tramadol) and major opioids (e.g., morphine and fentanyl) are utilized for refractory or severe pain. A combination of these pharmacological approaches is essential for optimal pain control and quality of life improvement. Radiotherapy when combined with adequate pain management, can lead to significant pain reduction [18].

The aim of this study is to evaluate the clinical value of combining RT with CDK4/6 inhibitors in terms of analgesic efficacy for bone metastases pain management, safety, and long-term clinical outcomes.

## 2. Materials and Methods

### 2.1. Study Population

Patients with histologically confirmed metastatic breast cancer, aged over 18 years, presenting with symptomatic bone metastases or at risk of skeletal complications, who received RT and concurrent CDK4/6 inhibitor therapy within the prospective observational COMBART (“Concomitant Radiotherapy and new drugs in Metastatic Breast cancer”) trial, were included in this analysis. COMBART is a single-center prospective observational registry which includes 4 cohorts of patients according to the drugs delivered concurrently: ADCs (cohort 1), anti-CDK4/6 (cohort 2), anti-HER2 (cohort 3) and immunotherapy (cohort 4). The primary aim of this analysis is to analyze pain response and toxicity during bone RT combined with anti-CDK4/6, while secondary outcomes are radiological response after SBRT and time to systemic treatment change. This study was conducted in accordance with the Declaration of Helsinki. COMBART TRIAL was approved by the Institutional Review Board of Fondazione Policlinico Campus Bio-Medico (protocol code approval number PAR 73.23 OSS and date of approval 17 May 2023). Informed consent was obtained from all patients involved in this study.

### 2.2. Radiotherapy

Radiotherapy was administered using moderate hypofractionated palliative RT, mainly used for the treatment of multiple adjacent lesions or extensive skeletal involvement or stereotactic body radiotherapy (SBRT) for localized bone lesions, delivered in 3–5 fractions with an 80% isodose prescription. SBRT was the treatment of choice for patients with oligoprogressive disease, with or without associated pain. All moderate hypofractionated RT treatments were delivered using volumetric arc therapy (VMAT).

### 2.3. CDK4/6 Inhibitor Therapy

Palbociclib was given at the dose of 125 mg per day for 3 weeks on, one week off. Some patients received a reduced dose of 100 mg per day in case of reported former toxicity. The dose was given in combination with letrozole in first line treatment or fulvestrant in women with disease progression following endocrine therapy. Premenopausal patients also received Ovarian Function Suppression (OFS) by luteinizing hormone-releasing hormone-agonists (LHRH-agonists). Ribociclib was given at the dose of 600 mg per day for 3 weeks on, one week off in combination with letrozole, and abemaciclib was administered in combination with letrozole or fulvestrant at the standard dose of 150 mg twice daily, with the dose being reduced to 100 mg twice daily or 50 mg twice daily when certain toxicities occurred. 

### 2.4. Outcome Measures

Pain was assessed using the numerical rating scale (NRS) before and after treatment. Mean ± SD values were calculated. Analgesic therapy was classified as none, NSAID-based, minor opioid-based, or major opioid-based. RT-related toxicities were categorized according to CTCAE v5.0. The net clinical benefit (NCB) was calculated as follows: % pain reduction (% patients with reduced NRS scores) − % patients developing any grade of toxicity. Radiological response in SBRT-treated patients was assessed using PET/CT scans with PERCIST criteria (version 1.0), classifying responses as complete metabolic response (CMR), partial metabolic response (PMR), stable metabolic disease (SMD), or progressive metabolic disease (PMD).

### 2.5. Statistical Analysis

Pain changes were analyzed with respect to the RT technique and CDK4/6 inhibitor therapy using a paired *T*-test. Changes in analgesic therapy post-RT were dichotomized in no change or improvement vs. worsening and differences by groups were evaluated by means of chi-square test. Kaplan–Meier analysis was used to evaluate the duration of CDK4/6 inhibitor therapy post-RT. Overall treatment time was analyzed from the initiation of CDK4/6 inhibitor therapy until treatment modification, whereas time to treatment change was evaluated from the completion of radiotherapy until treatment modification. A statistical significance level was set at *p* < 0.05.

## 3. Results

### 3.1. Patients Population

Forty patients received a total of 138 RT treatments. Patient characteristics are summarized in Table 1.

Moderate hypofractionated radiotherapy delivered by means of volumetric modulated arc therapy (VMAT) was used in 38 (23.7%), and SBRT in 100 patients (76.3%). Within the VMAT treatments, the following dose regimens were administered: 8 Gy in a single fraction (4 patients, 10.5%), 20 Gy in 5 fractions (6 patients, 15.8%), and 30 Gy in 10 fractions (28 patients, 76.7%).

For SBRT-treated patients, the following dose regimens were used: 21 Gy in 3 fractions (47 patients, 47%), 25 Gy in 5 fractions (4 patients, 4%), 35 Gy in 5 fractions (16 patients, 16%), 40 Gy in 5 fractions (6 patients, 6%), 30 Gy in 5 fractions (18 patients, 18%), and 27 Gy in 3 fractions (7 patients, 7%). In total, 1 patient (1.0%) received 20 Gy in 5 fractions with a simultaneous integrated boost (SIB) to 25 Gy to the GTV and another patient (1.0%) 24 Gy in 4 fractions.

The CDK4/6 inhibitors used included palbociclib (30.0%), ribociclib (50.0%), and abemaciclib (20.0%), administered in combination with standard endocrine therapy (letrozole or fulvestrant).

The median duration of CDK4/6 inhibitor treatment was 13.4 months (95% CI: 8.54–18.25), higher in SBRT patients (15.7 months vs. 9.77 months, *p* = 0.0168) compared to patients treated with moderate hypofractionation.

### 3.2. Pain Assessment

The baseline mean NRS score was 3.5 ±2.9. The NRS score was higher in patients undergoing moderate hypofractionated RT (mean value 5.39, SD 2.4 vs. 2.79, SD 2.9; *p* < 0.001). The combined therapy resulted in a significant reduction in pain, as evidenced by the decrease in the mean NRS score from 3.52 (pre-treatment) to 1.31 (post-treatment) (*p* < 0.001). The reduction in the mean NRS was greater in patients treated with moderate hypofractionation (58.6% vs. 39.9%; *p* = 0.016). Pain reduction was independent of the type of CDK4/6 inhibitor used: palbociclib vs. others (−2.31 vs. −2.18), ribociclib vs. others (−1.98 vs. −2.39), abemaciclib vs. others (−2.38 vs. −2.18).

A total of 26 patients (65%) had baseline analgesic therapy. Of these, 51.2% were treated with NSAIDs, 4.8% with minor opioids, and 17.6% with major opioids.

A reduction in analgesic therapy was observed in 3 patients (14.3%), stability in 17 patients (70.6%), and an increase in 2 patients (10.3%). The reduction in pharmacologic analgesic therapy was more frequently observed in patients treated with palbociclib (35.4%) compared to those treated with ribociclib (11.0%) and abemaciclib (0.0%) (*p* = 0.001).

### 3.3. Toxicity

Toxicity was predominantly Grade 1–2 and easily manageable. A total of eight toxicities (6.1%) were recorded, four hematologic (3.05%) and four non-hematologic (3.05%) events (see Table 2).

The non-hematologic toxicities included dysphagia (n = 3) and nausea (n = 1). Grade 2 toxicities were observed in two cases (1.5%), both being represented by neutropenia.

### 3.4. Net Clinical Benefit

The net clinical benefit (NCB) for the entire study population was as follows: NCB = 0.66 − 0.06 = 0.6. The NCB in patients undergoing radiotherapy (RT) with moderate hypofractionated radiotherapy was = 0.90 − 0.16 = 0.74. The NCB in patients undergoing stereotactic body radiotherapy (SBRT) was = 0.57 − 0.04 = 0.52.

### 3.5. SBRT Response

The response was assessable in 83 out of 100 lesions treated with SBRT, for which pre- and post-radiotherapy (RT) PET-CT imaging was available. The complete response (CR) rate was 43/83 (51.8%), the partial response (PR) rate was 34/83 (40.96%), and the stable disease (SD) rate was 6/83 (7.22%). The overall response rate (CR + PR) was 77/83 (92.77%), while the clinical benefit rate (CR + PR + SD) was 100%.

Radiological complete response was more frequently achieved in patients undergoing SBRT + ribociclib compared to those receiving SBRT + palbociclib or SBRT + abemaciclib (72.5% vs. 60.0% and 60.7%, *p* = 0.001).

In our cohort, no differences were observed in terms of pain response between patients who achieved a complete radiological response and those who did not.

## 4. Discussion

Although previous studies have explored the safety and feasibility of combining RT with CDK4/6 inhibitors in patients with bone only metastatic breast cancer [19,20] to the best of our knowledge, this is the first study specifically addressing the clinical benefit of RT in terms of pain relief and changes in analgesic therapy in this patients population. The rationale for combining RT with CDK4/6 inhibitors, despite the availability of other treatment strategies, lies in the complementary mechanisms and clinical objectives of the two modalities. Radiotherapy offers rapid, localized pain relief and disease control, which is particularly valuable in symptomatic patients or in the context of oligoprogression [11,12,13,21]. Meanwhile, CDK4/6 inhibitors provide sustained systemic control in HR+/HER2− metastatic breast cancer [5,6,7,8,9]. This study aims to offer new insights into the symptomatic management of bone metastases in the era of CDK4/6 inhibitors. The aim of our analysis was to evaluate the role of RT in pain control when combined with CDK4/6 inhibitors. A significant pain reduction was observed following RT (*p* < 0.01) and CDK4/6 inhibitors, and also the net clinical benefit (NCB) was positive (value = 0.6), with a favorable efficacy/toxicity profile, regardless of the type of CDK4/6 inhibitor used.

The 2024 ASTRO guidelines on the RT management of bone metastases recommend as treatment for symptomatic bone metastases the use of RT regimens such as 8 Gy in a single fraction or 20 Gy in 5 fractions and/ or 30 Gy in 10 fractions [22]. In terms of pain remission, both single-fraction and multi-fraction regimens are equivalent, with 30-day response rates between 49% and 88%, and 3-month rates between 60% and 74%.

Recently, a systematic review of the literature [23] including 18 studies with a total of 1685 patients showed that overall pain response was not significantly different between hypofractionated RT and SBRT at 1 (RR 1.14; 95% CI, 0.99–1.30), 3 (RR 1.19; 95% CI, 0.96–1.47), and 6 (RR 1.22; 95% CI, 0.96–1.54) months. However, a complete pain response was higher in patients treated with SBRT [23,24]. Therefore, for selected patients with good performance status (ECOG 0–2) and no neurological symptoms, the guidelines conditionally recommend SBRT over conventional hypofractionated RT, especially in cases of oligometastatic disease [22].

In our case series, pain reduction was greater in patients treated with hypofractionated RT compared to those treated with SBRT (58.6% vs. 39.9%; *p* = 0.016), with a higher NCB compared to SBRT (0.74 vs. 0.52). The greater benefit observed may be partially due to the higher baseline pain of these patients compared to those treated with SBRT (mean score 5.39, SD 2.4 vs. 2.79, SD 2.9; *p* < 0.001), as well as the fact that SBRT treatments represented a minority of the total treatments included in the analysis (31/131, 23.66%).

Overall, 14.3% of patients reduced their ongoing pharmacological therapy for pain control. In particular, analgesic therapy reduction was more commonly observed in patients receiving palbociclib (35.4%) compared to those on ribociclib (11.0%) and abemaciclib (0.0%) (*p* = 0.001).

Although all three CDK4/6 inhibitors have been shown to act as radiosensitizers in vitro and in vivo [25], we lack direct comparative studies, and the differing pharmacokinetics of the molecules could underlie these results, perhaps reflecting a different synergy in pain signaling.

Moreover, among patients treated with SBRT, a CMR was more frequently achieved in those receiving SBRT + ribociclib compared to SBRT + palbociclib or abemaciclib (72.5% vs. 60.0% and 60.7%, respectively; *p* = 0.001). This could be related to the different effects of the three inhibitors on the tumor bone microenvironment [26].

Overall, RT treatments were well tolerated, with an overall complication rate of 6.1%, grade 2 toxicity (G2) at 1.56%, and no grade 3 (G3) toxicities. These toxicity rates are lower than those reported in the meta-analysis by Becherini et al. [17], which documented an overall G3 toxicity rate of 22%, including G3 hematologic toxicity rates as high as 14%.

We believe that the lower toxicity rate observed in our study may be attributed to several factors. First, our case series predominantly includes stereotactic treatments (74.28%), which are typically short in duration and associated with lower rates of acute toxicity [27]. Similarly, Kubeczko et al., in a single-institution experience involving 34 patients treated with SBRT and CDK4/6 inhibitors across 44 metastatic sites (70.4% bone lesions), reported two cases of grade 3 neutropenia and one case of grade 4 neutropenia, for an overall rate of 8.8% [19]. Second, in our prospective study, VMAT was systematically employed to optimize dose distribution and minimize radiation exposure to organs at risk (OARs), which likely contributed to the favorable safety profile observed.

Although it was not the primary objective of this study, an interesting finding was the time to drug change after radiotherapy. The median duration of CDK4/6 inhibitor therapy following radiotherapy was 13.4 months (95% CI: 8.54–18.25), and it was longer in patients who received SBRT (15.7 months vs. 9.77 months; *p* = 0.0168).

This is notable because it highlights the role of radiotherapy not only as a symptomatic treatment but also as a means of managing oligoprogression and delaying systemic therapy changes.

In this context, recent results from the AVATAR study [28] showed that SBRT, in a population of 32 patients with oligoprogressive lesions, is an excellent strategy for maintaining systemic therapy with CDK4/6 inhibitors in metastatic disease, achieving a median time to systemic therapy change of 10.4 months, with 46% of patients remaining on CDK4/6 inhibitors for more than 6 months.

Finally, more recently, Kubeczko et al. [20], in a large cohort of 114 patients undergoing bone RT in combination with CDK4/6 inhibitors, emphasized that the addition of radiotherapy in patients with bone disease was associated with improved outcomes, although the results were not statistically significant.

From a clinical perspective, the results of this study, including preliminary results, support the integration of RT in the treatment of patients with HR+/HER2− metastatic breast cancer receiving CDK4/6 inhibitors. The significant pain reduction and the potential to reduce analgesic therapy with the limited toxicity profile observed suggest that RT can be safely administered without the need to discontinue systemic therapy. These findings provide valuable insights for multidisciplinary teams, offering practical evidence to support the safe and effective integration of local and systemic therapy. This is particularly relevant for symptomatic patients, whose management should not be limited to palliative treatments only, but should aim to achieve the best possible clinical outcomes.

This study has several limitations. First, the relatively small sample size limits the generalizability of the findings and reduces the possibility to detect differences in subgroups. Second, the observational design, although prospective, may be subject to selection bias and confounding factors. Third, the heterogeneity in radiotherapy techniques and dose regimens, as well as the use of different CDK4/6 inhibitors, may have affected treatment response and toxicity outcomes. Further prospective studies with larger, homogeneous cohorts are needed to validate these findings.

## 5. Conclusions

In conclusion, our prospective study appears to support the combination of bone RT with CDK4/6 inhibitors being associated with a favorable net clinical benefit in reducing bone metastases related pain. Even if the results should be interpreted as preliminary and hypothesis generating, the toxicity was manageable, allowing for the continuation of systemic therapy without interruption during RT course. Notably, although SBRT did not result in better pain control, it may represent a useful strategy in selected patients with bone oligoprogression, potentially delaying the time to treatment change. These findings offer meaningful insights for clinical decision making and underscore the importance of multidisciplinary strategies in this setting.

## Figures and Tables

**Table 1:** Patient and treatment characteristics.

	Patients (n, %)	RT Treatment (Number, %)	RT Technique (SBRT, VMAT)
Total	40 (100%)	138 (100%)	SBRT: 100 (76.4%) VMAT: 38 (23.6%)
Age (years)			
Mean (SD)	60 (12)		
Menopausal Status			
Premenopausal or Perimenopausal	11 (27.5%)	32 (23.2%)	SBRT: 21 (65.6%) VMAT:11 (34.4%)
Postmenopausal	29 (72.5%)	106 (76.8%)	SBRT: 79 (74.5%) VMAT: 27 (25.5%)
CDK4/6 Inhibitor			
Palbociclib	12 (30%)	41 (29.7%)	SBRT: 27(65.8%) VMAT: 14 (34.2%)
Abemaciclib	8 (20%)	29 (21.0%)	SBRT: 20 (68.9%) VMAT: 9 (31.1%)
Ribociclib	20 (50%)	68 (49.3%)	SBRT: 53 (77.9%) VMAT: 15 (22.1%)
Line of Treatment			
First	36 (90%)	119 (86.3%)	SBRT: 92 (77.3%) VMAT: 27 (22.7%)
Second	4 (10%)	19 (13.7%)	SBRT: 8 (42.1%) VMAT: 11 (57.9%)
Setting			
Endocrine Sensitive	27 (67.5%)	98 (71.0%)	SBRT: 73 (74.5%) VMAT: 25 (25.5%)
Endocrine Resistant	13 (32.5%)	40 (29.0%)	SBRT: 27 (67.5%) VMAT: 13 (32.5%)
Endocrine Therapy			
Aromatase Inhibitor	17 (42.5%)	57 (41.3%)	SBRT: 41 (71.9%) VMAT: 16 (28.1%)
Fulvestrant	23 (57.5%)	81 (58.7%)	SBRT: 59 (72.8%) VMAT: 22 (27.2%)
Comorbidity			
No	26 (65%)	92 (66.7%)	SBRT: 65 (70.6%) VMAT: 27 (29.4%)
Yes	14 (35%)	46 (33.3%)	SBRT: 35 (76.1%) VMAT: 11 (23.9%)
Analgesic Therapy			
No	14 (35%)	37 (26.8%)	SBRT: 31 (70.6%) VMAT: 6 (29.4%)
Yes	26 (65%)	101 (73.2%)	SBRT: 69 (68.3%) VMAT: 32 (31.7%)
Pain NRS score			
Pre-RT (mean, SD)	3.5 (2.9)		
Post-RT (mean, SD)	1.2 (1.6)		

Abbreviations: RT = radiotherapy; SBRT = stereotactic body radiotherapy; VMAT = volumetric modulated arc therapy; NRS: numerical rating scale.

**Table 2:** Toxicity description.

Patient	Toxicity Type (CTCAE GRADE)	Radiotherapy Technique	Total Dose/Dose per Fraction (Gy/cGy)	RT Site	CDK4/6 Inhibitor
1	Neutropenia (G2)	VMAT	30/300	Lumbar spine (L3–L5)	Palbociclib
2	Neutropenia (G2)	SBRT	24/600	Right sacral ala	Ribociclib
3	Neutropenia (G1)	VMAT	30/300	Sacrum and bilateral sacral alae	Abemaciclib
4	Anemia (G1)	VMAT	30/300	Lumbar spine (L4–L5)	Palbociclib
5	Nausea (G1)	SBRT	20/400	Thoracic spine (T3–T5)	Abemaciclib
6	Dysphagia (G1)	VMAT	30/300	Cervical spine (C1–C3)	Ribociclib
7	Dysphagia (G1)	VMAT	30/300	Cervical spine (C1)	Abemaciclib
8	Dysphagia (G1)	SBRT	21/700	Thoracic spine (T3)	Ribociclib

Abbreviations: RT = radiotherapy; SBRT = stereotactic body radiotherapy; VMAT = volumetric modulated arc therapy.

## Data Availability

Data are available upon reasonable request from the corresponding author.

## Abbreviations

The following abbreviations are used in this manuscript:

CDK4/6 inhibitor	Cyclin-dependent kinase 4 and 6 inhibitors
ET	Endocrine therapy
HR+ BC	Hormone Receptor-Positive Breast Cancer
RT	Radiotherapy
SBRT	Stereotactic body radiation therapy
NSAIDs	Nonsteroidal anti-inflammatory drugs
NCB	Net clinical benefit
VMAT	Volumetric modulated arc therapy
CMR	Complete metabolic response
PMR	Partial metabolic response
SMD	Stable metabolic disease
PMD	Progressive metabolic disease
